# Cell Death in the Epithelia of the Intestine and Hepatopancreas in *Neocaridina heteropoda* (Crustacea, Malacostraca)

**DOI:** 10.1371/journal.pone.0147582

**Published:** 2016-02-04

**Authors:** Lidia Sonakowska, Agnieszka Włodarczyk, Grażyna Wilczek, Piotr Wilczek, Sebastian Student, Magdalena Maria Rost-Roszkowska

**Affiliations:** 1 University of Silesia, Department of Animal Histology and Embryology, Bankowa 9, 40–007, Katowice, Poland; 2 University of Silesia, Department of Animal Physiology and Ecotoxicology, Bankowa 9, 40–007, Katowice, Poland; 3 Heart Prosthesis Institute, Bioengineering Laboratory, Wolnosci 345a, 41–800, Zabrze, Poland; 4 Faculty of Automatic Control, Electronics and Computer Science, Silesian University of Technology, Akademicka 16, 44–100, Gliwice, Poland; Columbia University, UNITED STATES

## Abstract

The endodermal region of the digestive system in the freshwater shrimp *Neocaridina heteropoda* (Crustacea, Malacostraca) consists of a tube-shaped intestine and large hepatopancreas, which is formed by numerous blind-ended tubules. The precise structure and ultrastructure of these regions were presented in our previous studies, while here we focused on the cell death processes and their effect on the functioning of the midgut. We used transmission electron microscopy, light and confocal microscopes to describe and detect cell death, while a quantitative assessment of cells with depolarized mitochondria helped us to establish whether there is the relationship between cell death and the inactivation of mitochondria. Three types of the cell death were observed in the intestine and hepatopancreas–apoptosis, necrosis and autophagy. No differences were observed in the course of these processes in males and females and or in the intestine and hepatopancreas of the shrimp that were examined. Our studies revealed that apoptosis, necrosis and autophagy only involves the fully developed cells of the midgut epithelium that have contact with the midgut lumen–D-cells in the intestine and B- and F-cells in hepatopancreas, while E-cells (midgut stem cells) did not die. A distinct correlation between the accumulation of E-cells and the activation of apoptosis was detected in the anterior region of the intestine, while necrosis was an accidental process. Degenerating organelles, mainly mitochondria were neutralized and eventually, the activation of cell death was prevented in the entire epithelium due to autophagy. Therefore, we state that autophagy plays a role of the survival factor.

## Introduction

In multicellular organisms the processes of programmed cell death (PCD) are connected with physiological and pathological alterations of cells that cause their deletion from tissues and organs. Therefore, it plays an important role in maintaining tissue homeostasis [1]. The relationship between the proliferation of cells and their death can regulate cell number, their proper functioning and eventually the development, differentiation and growth of tissues [2]. Among the types of programmed cell death, apoptosis, which can be caused by many factors (e.g., xenobiotics, pathogens, starvation, irradiation) [3,4], has been recognized. It is not combined with inflammatory reactions, which can occur when the apoptotic cell cannot be discharged from the tissue and thus activate inflammation. Another type of cell death is necrosis, which can be caused by mechanical damages (passive process) or can be non-apoptotic programmed cell death, which is called paraptosis [5,6]. There are many differences in the course of these processes that are connected with the transformation of mitochondria, cytoplasmic vacuolation, alterations in nuclei and DNA, etc. [5]. Additionally, in response to starvation and various stressors, autophagy can be activated in order to degrade and/or exploit the reserve material, toxins or pathogens in order for the cell to survive. During this process, long-lived proteins and organelles are delivered to autophagosomes and digested inside autolysosmes. Unchecked autophagy can eventually cause cell death. Autophagy is a rather non-selective process. However, it can become selective when specific organelles are targeted into autophagosomes [7]. Therefore, the selective organelles can be enclosed and degraded inside autophagosomes–mitochondria (mitophagy), cisterns of endoplasmic reticulum (reticulophagy), lipids (lipophagy), fragments of the nucleus (nucleophagy), etc. [8].

Mitochondria are organelles that are essential for the production of energy which must be delivered to all of the organelles in order to perform different functions in a cell. There is evidence that mitochondria are also involved in cell death [9,10]. They can activate apoptosis by releasing apoptogenic factors [11], which activate the downstream execution phase of apoptosis. Therefore, measurements of changes in the mitochondrial potential (ΔΨm) can show physiological condition of cells and tissues [12]. The above-mentioned types of cell death can run parallel in the cell, or can follow one another other. The epithelia of the digestive system in invertebrates, which plays a strategic role in digestion and detoxification, are treated as the good models for the analysis of the pathways of cell death. During our previous studies on the midgut of the freshwater shrimp *Neocaridina heteropoda* (Crustacea, Malacostraca) [13], we noted the appearance of autophagy, apoptosis and necrosis. The natural environment and feeding habitats of this species are similar to that observed in freshwater crustaceans common for fauna all over the world. Additionally, *N*. *heteropoda* is widely available and bred, easy to possess and breed in the laboratory conditions. Therefore, the aim of the present study was to describe processes of the cell death with an emphasis on the differences between the intestine and hepatopancreas (two organs that form the midgut of *N*. *heteropoda*) and between the males and females of the species that were examined. Additionally, we compared the percentage of cells that had signs of the death together with the number of mitochondria that had their potential changed. Among arthropods, processes of the cell death in the midgut epithelium are mainly described for Hexapoda, while information connected with Crustacea presents only its basic ultrastructure. In the literature, we may find the data that many stressors [3,4], e.g. toxic substances (pesticides or metal ions) can be dissolved in water and can originate from the soil, air or even plants. Therefore, the relationship between autophagy, apoptosis and necrosis are important aims of each analysis of the cell death. In addition, *N*. *heteropoda* belongs to Malacostraca, the largest class of Crustacea. This group of Hexapoda contains animals which have colonized marine, freshwater and terrestrial environments. so they can be exposed to different stressors. Knowledge about the course of cell death will help in elucidation how crustaceans can oppose them. In most cases, freshwater organisms are sensitive to these substances, so they seem to be good models for studies the cell death. They are also sensitive to long periods of starvation [4]. Therefore, the results may be helpful during our further studies, which will be connected with activation of cell death and changes in the mitochondrial potential due to external stressors or starvation. The results of studies on the midgut epithelium in males and females of *N*. *heteropoda*, which were not exposed to any stressors, could be treated as the control group.

## Results

The midgut of *Neocardina heteropoda* is formed by two distinct organs–the intestine and hepatopancreas. The intestine is a tube-shaped organ with an epithelium that is composed of D-cells (digestive cells) and R-cells (regenerative cells), which are only accumulated in the anterior region of the intestine. The hepatopancreas is formed by numerous tubules and three zones were distinguished along the length of each tubule–the distal zone with R-cells, the medial zone with differentiating cells and finally, the proximal zone with F-cells (fibrillar cells) and B-cells (storage cells). The precise structure and ultrastructure of the intestine and hepatopancreas were presented in our previous article [13]. Autophagy, apoptosis and necrosis were noted in the midgut of shrimp that were analyzed. Among these types of the cell death, autophagy was the dominant process that was observed in the majority of epithelial cells, while apoptosis and necrosis occurred only occasionally (Tables 1–3). No differences were observed between males and females; therefore, the results presented below relate to both sexes.

**Table 1:** Percentage [%] of cells in the entire intestine and proximal zone of the hepatopancreatic epithelium in *N*. *heteropoda* that showed signs of autophagy.

	all cells	cells with no signs of autophagy	autophagic cells	cells with no signs of autophagy [%]	autophagic cells [%]
**intestine 1**	152	92	60	60.53	39.47
**intestine 2**	220	129	91	58.64	41.36
**intestine 3**	231	138	938	59.74	40.26
**hepatopancreas 1**	264	167	97	63.26	36.74
**hepatopancreas 2**	400	263	137	65.75	34.25
**hepatopancreas 3**	242	151	91	62.40	37.60

**Table 2:** Percentage [%] of apoptotic cells in the anterior region of the intestine and proximal zone of hepatopancreatic epithelium in *N*. *heteropoda*.

	all cells	not apoptotic cells	apoptotic cells	not apoptotic cells [%]	apoptotic cells [%]
**intestine 1**	373	276	97	74.00	26.00
**intestine 2**	353	273	80	77.34	22.66
**intestine 3**	418	325	93	77.75	22.25
**hepatopancreas 1**	104	85	19	81.74	18.26
**hepatopancreas 2**	126	103	23	81.75	18.25
**hepatopancreas 3**	208	171	37	82.22	17.78

**Table 3:** Percentage [%] of necrotic cells in the entire intestine and proximal zone of the hepatopancreatic epithelium in *N*. *heteropoda*.

	all cells	not necrotic cells	necrotic cells	not necrotic cells [%]	necrotic cells [%]
**intestine 1**	152	144	8	94.74	5.26
**intestine 2**	220	211	9	95.90	4.10
**intestine 3**	231	219	12	94.80	5.20
**hepatopancreas 1**	264	260	4	98.48	1.52
**hepatopancreas 2**	400	390	10	97.50	2.50
**hepatopancreas 3**	242	238	4	98.35	1.65

### Autophagy

Autophagy (macroautophagy) occurred in the cytoplasm of the D-cells in the intestine epithelium (about 40% of the cells showed signs of autophagy) and in the cytoplasm of F- and B-cells in the proximal zone of the hepatopancreatic tubules (about 36% of the cells showed signs of autophagy) (Table 1). The cytoplasm of the R-cells in both, the intestine and hepatopancreas, did not show any signs of autophagy.

The following description of autophagy relates to both organs–the intestine and hepatopancreas as no differences were observed, despite some of the differences that are presented below.

**Fig 1:**
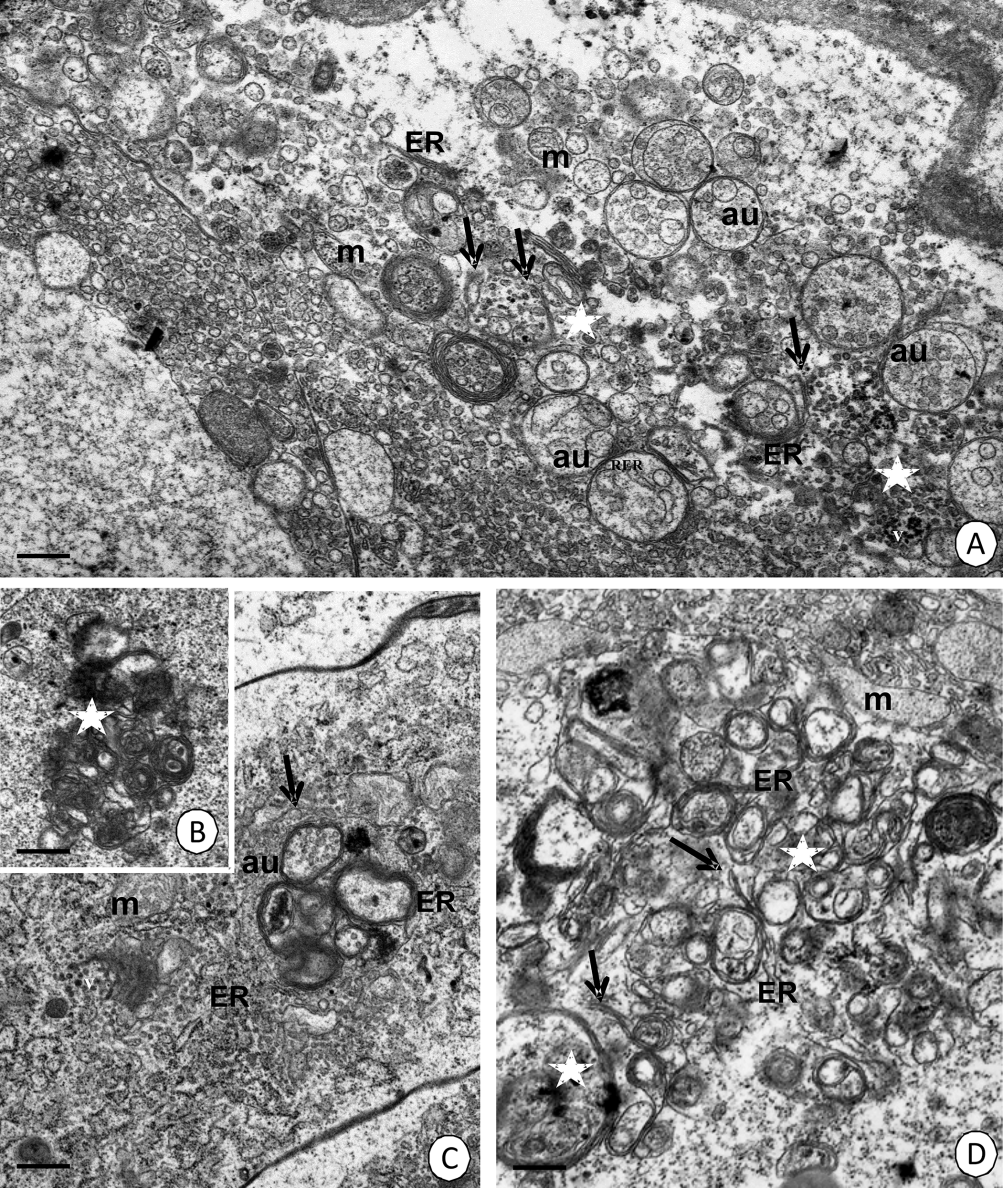
Autophagosomes formation in the *N*. *heteropoda* midgut. **(A)** Cells of the proximal zone of the hepatopancreatic tubules. TEM. Bar = 0.55 μm. **(B-C)** Intestine digestive cells that showed signs of autophagy. TEM. **(B)** Bar = 0.4 μm. **(C)** Bar = 0.5 μm. **(D)** Hepatopancreatic epithelial cell that showed signs of autophagy. TEM. Bar = 0.45 μm. Cisterns of the endoplasmic reticulum (ER), mitochondria (m), vesicles with electron-dense content (v), autophagosomes (au), agglomerations of degenerating organelles (asterisks), phagophore formation (arrows).

**Fig 2:**
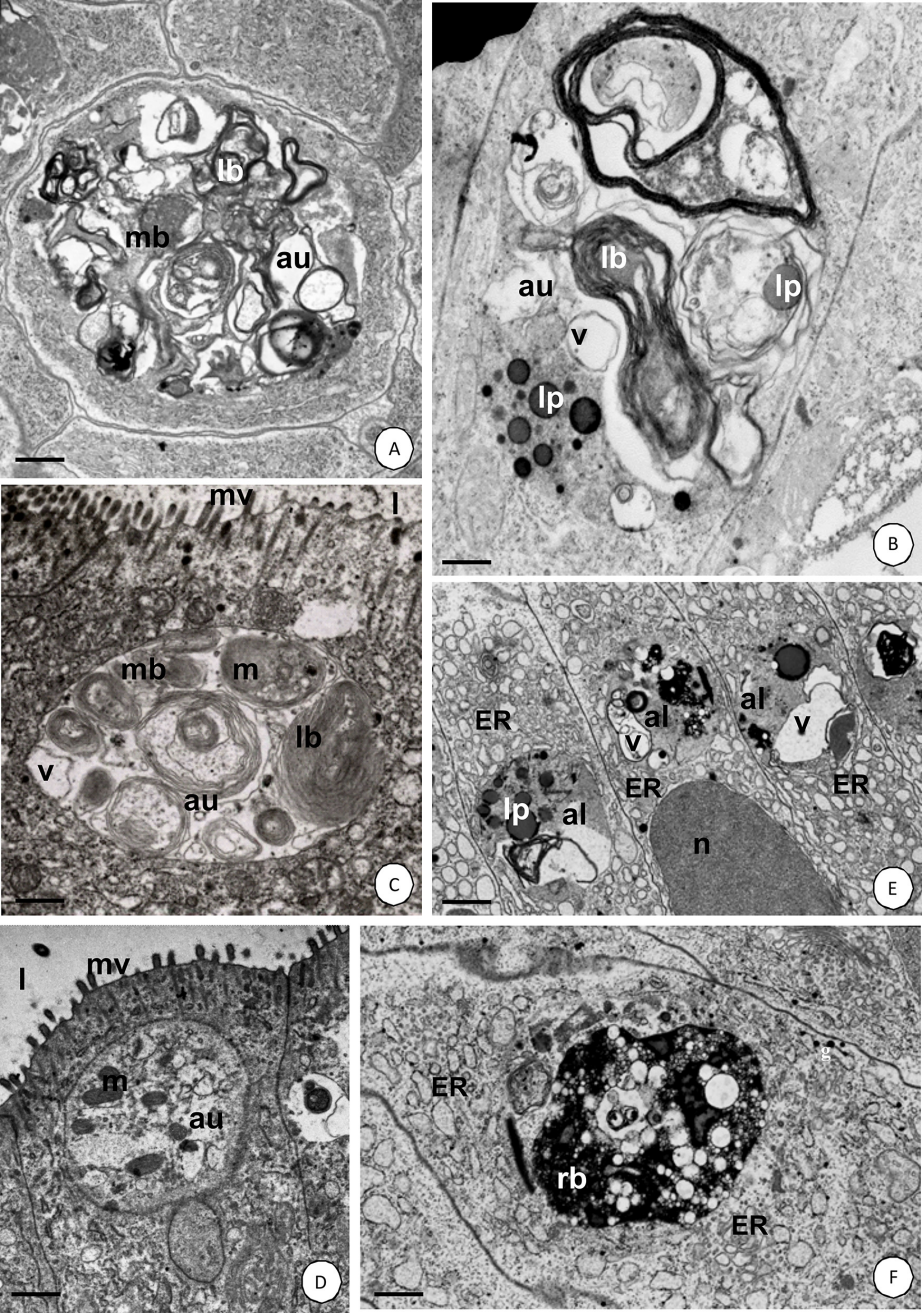
Autophagy in the *N*. *heteropoda* midgut. **(A)** Hepatopancreas. TEM. Bar = 0.15 μm. **(B-D)** Intestine digestive cells. **(B)** TEM. Bar = 0.1 μm. **(C)** Mitophagy. TEM. Bar = 0.15 μm. **(D)** Mitophagy. TEM. Bar = 0.55 μm. **(E-F)** Hepatopancreas. **(E)** TEM. Bar = 0.75 μm. **(F)** TEM. Bar = 0.45 μm. Autophagosomes (au), autolysosomes (al), residual body (rb), cisterns of the endoplasmic reticulum (ER), mitochondria (m), microvilli (mv), midgut lumen (l), vacuoles (v), lamellar bodies (lb), lipids (l).

Transformed and degenerating organelles formed distinct agglomerations (Fig 1A and 1B), which were gradually surrounded by a double-membraned structure that is called a phagophore, and is formed by cisterns of the endoplasmic reticulum (Fig 1A, 1C and 1D). The membrane of the phagophore expanded due to its fusion with numerous small vesicles and gradually enclosed further organelles. Eventually, the ends of the phagophore connected and the autophagosome was formed (Fig 2A and 2B). Cisterns of the endoplasmic reticulum, vesicles that had an electron-lucent content, vacuoles, multivesicular bodies, lamellar bodies, lipids and electron-dense granules could be observed inside autophagosomes (Fig 2B and 2C). However, degenerated mitochondria were mainly accumulated in the autophagosomes (mitophagy) (Fig 2C and 2D). The autophagosomes fused with electron-dense lysosomes and autolysosomes were formed (Fig 2E). The digestion of the autolysosomal interior caused the formation of residual bodies (Fig 2F). Eventually, the residual bodies were discharged into the midgut lumen in a manner that resembled apocrine secretion. Therefore, the apical cell membrane formed an evagination into the intestinal or hepatopancreatic lumen and the cytoplasm with some organelles and autophagosomes/autolysosmes/residual bodies penetrated the evagination. The evagination was finally separated from the entire cell and the autophagosomal structures were digested inside the midgut lumen (not shown).

**Fig 3:**
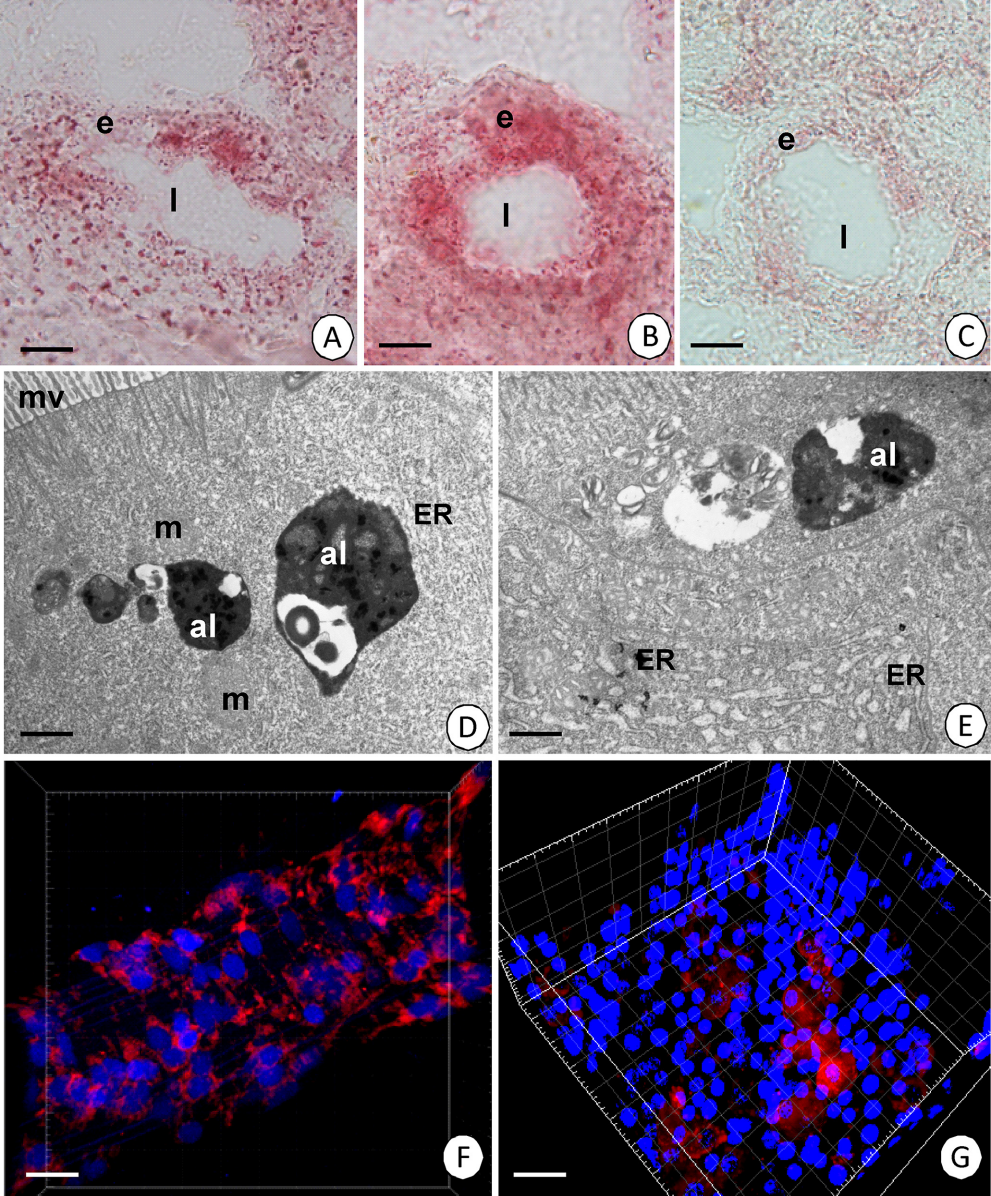
Autolysosomes and acid phosphatase localization in the *N*. *heteropoda* midgut. **(A)** Transverse section through the intestine with pink spots of acid phosphatase localization. Midgut lumen (l), midgut epithelium (e). Acid phosphatase staining. Light microscope. Bar = 20 μm. **(B)** Transverse section through the hepatopancreatic tubule with pink spots of acid phosphatase localization. Midgut lumen (l). Acid phosphatase staining. Light microscope. Bar = 30 μm. **(C)** Transverse section through the intestine. Negative control for acid phosphatase staining. Midgut lumen (l) midgut epithelium (e). Light microscope. Bar = 19 μm. **(D)** Intestine. Electron-dense autolysosomes (al), mitochondria (m), cisterns of ER (ER), microvilli (mv). Acid phosphatase staining. TEM. Bar = 0.8 μm. **(E)** Hepatopancreas. Electron-dense autolysosomes (al), cisterns of ER (ER). Acid phosphatase staining. TEM. Bar = 0.8 μm. **(F)** 3D representation of the accumulation of lysosomes and autolysosomes (red signals). Nuclei (blue signals). A fragment of the intestine. LysoTracker Red and Hoechst 33342 staining. Confocal microscope. Bar = 10.5μm. **(G)** 3D representation of the accumulation of lysosomes and autolysosomes (red signals). Nuclei (blue signals). A fragment of the proximal zone of the hepatopancreas. LysoTracker Red and Hoechst 33342 staining. Confocal microscope. Bar = 10μm.

Autophagy was confirmed by acid phosphatase staining in order to investigate the material using the light (Fig 3A and 3B) and transmission electron microscopes (Fig 3C and 3D) and also by LysoTracker staining (Fig 3E and 3F, S1 Video, S2 Video).

### Apoptosis

**Fig 4:**
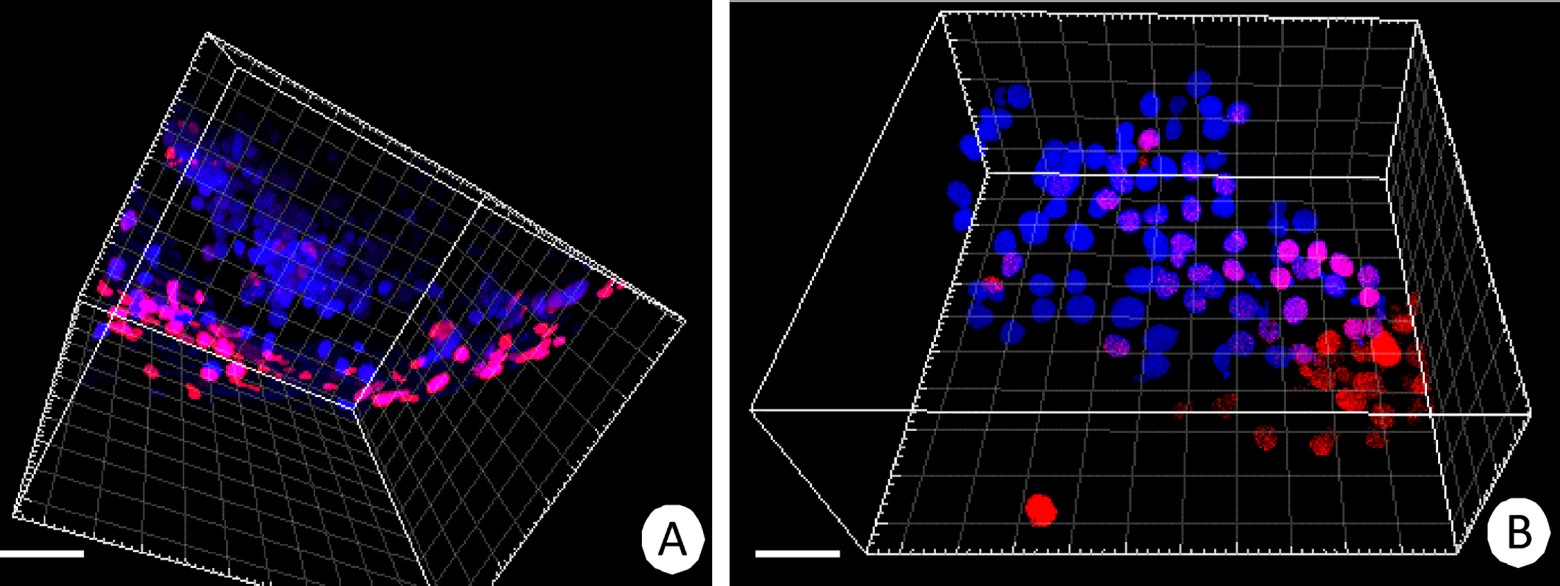
Apoptosis in the *N*. *heteropoda* midgut. 3D representation of the Tunel assay and Hoechst 33342 staining. Nuclei of apoptotic cells (red), nuclei (blue). Confocal microscope. **(A)** Fragment of the anterior region of the intestine. Bar = 9.5μm. **(B)** Fragment of the hepatopancreatic tubule. Bar = 11μm.

Apoptosis overlapped in the anterior region of the intestine. It concerned the digestive cells of intestine epithelium (D-cells) (about 23% of digestive cells in the anterior region of intestine showed signs of apoptosis) (Table 2), while the R-cells that accumulated in the anterior region of the intestine did not die in an apoptotic way (Fig 4A, S3 Video). In the case of the hepatopancreas, apoptosis was observed only in the proximal zone of hepatopancreatic tubules where F- and B-cells died in an apoptotic way (about 18% of epithelial cells in the proximal zone showed signs of apoptosis) (Table 2) (Fig 4B, S4 Video).

The following description of apoptosis relates to both organs–the intestine and hepatopancreas as no differences were observed.

**Fig 5:**
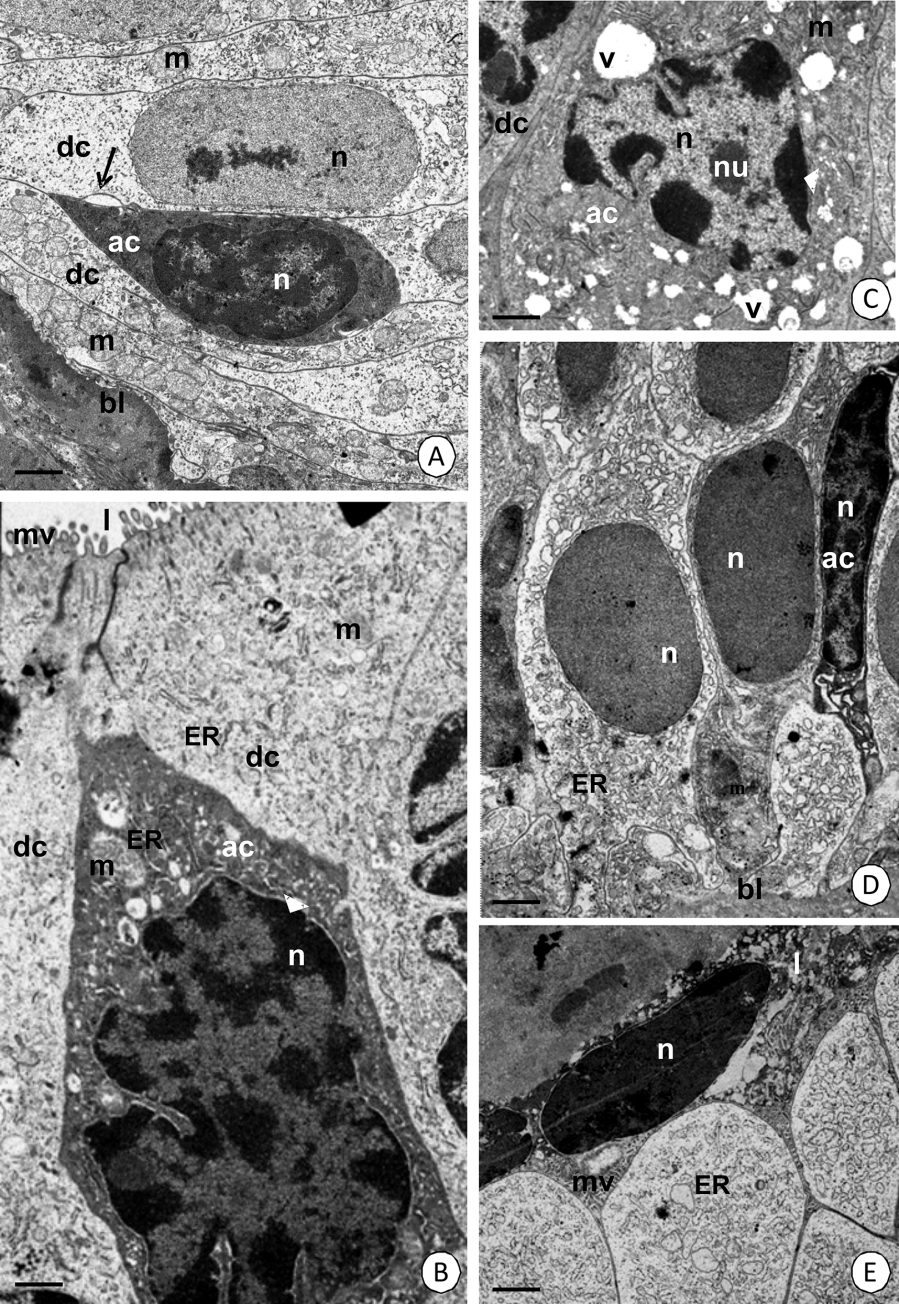
Ultrastructural features of apoptotic cells in the *N*. *heteropoda* midgut. **(A-C)** Intestine. TEM. **(A)** Electron-dense cytoplasm of an apoptotic cell (ac). Distinct extracellular spaces (arrows) between the apoptotic and neighboring digestive cells (dc). Bar = 1.7 μm. **(B-C)** Lobular shaped nucleus (n) with patches of heterochromatin. **(B)** Bar = 1 μm. **(C)** Bar = 1.2 μm. **(D-E)** Hepatopancreas. TEM. **(D)** Apoptotic cell (ac) losing contact with the basal lamina (bl). Bar = 1.5 μm. **(E)** Apoptotic cell (ac) discharged into the midgut lumen (l). Bar = 2 μm. Cisterns of ER (ER), vacuoles (v), mitochondria (m), nucleus (n), nucleolus (nu), basal lamina (bl).

The cell began to shrink at the beginning of apoptosis. The cytoplasm became electron-dense (Fig 5A and 5B) and distinct extracellular spaces appeared between the apoptotic cell and adjacent cells (Fig 5A). The nucleus of the apoptotic cell shrank and developed a lobular shape. Its chromatin formed electron-dense patches that gathered near the nuclear envelope (Fig 5B and 5C). Numerous mitochondria degenerated and lost their cristae. Cisterns of the rough and smooth endoplasmic reticulum became swollen (Fig 5B and 5D) and numerous vacuoles with an electron-lucent content occurred in the cytoplasm of the apoptotic cell (Fig 5C). The apoptotic cell gradually lost contact with the basal lamina (Fig 5D) and eventually the entire cell was discharged into the midgut lumen (Fig 5E, Table 4).

**Table 4:** Alterations of the cell and organelles during apoptosis and necrosis.

	Apoptosis	Necrosis
**Cell**	Shrinkage	swelling
**Cytoplasm**	electron-dense	electron-lucent
**Extracellular spaces**	+	-
**Nucleus**	lobular shape, shrinking	swollen
**Mitochondria**	Degenerating	degenerating
**Cisterns of ER**	Swollen	degenerating
**Vacuoles**	Few	numerous
**Apical cell membrane**	Undisturbed	disrupted
**Fate of a cell**	entire cell discharged into the midgut lumen	organelles released into the midgut lumen

### Necrosis

**Fig 6:**
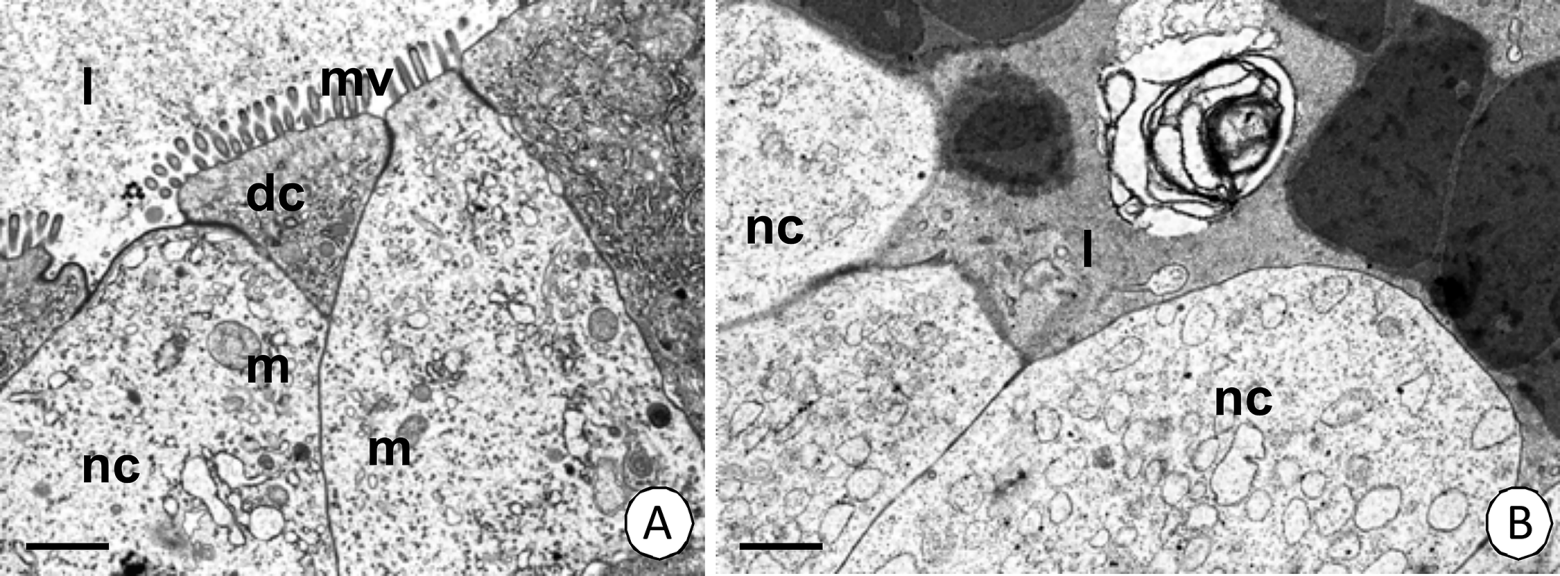
Necrosis in the *N*. *heteropoda* midgut. **(A)** Intestine. TEM. Bar = 1 μm. **(B)** Hepatopancreas. TEM. Bar = 0.9 μm. Necrotic cell (nc) with electron-lucent cytoplasm, midgut lumen (l), digestive cells (dc) of the intestine, mitochondria (m), microvilli (mv), cisterns of ER (ER).

Necrosis occurred occasionally in the epithelium of the intestine and proximal zone of hepatopancreatic tubules (about 4.8% of the epithelial cells of intestine and 1.9% of the epithelial cells of hepatopancreas showed signs of necrosis) (Table 3). The cytoplasm of a necrotic cell became electron-lucent and numerous vacuoles appeared (Fig 6A). The nucleus became swollen. The number of organelles decreased and they gradually degenerated. The apical cell membrane lost its microvilli (Fig 6B) and eventually the apical cell membrane broke. Organelles were released into the midgut lumen (Table 4).

### Mitochondrial potential

**Fig 7:**
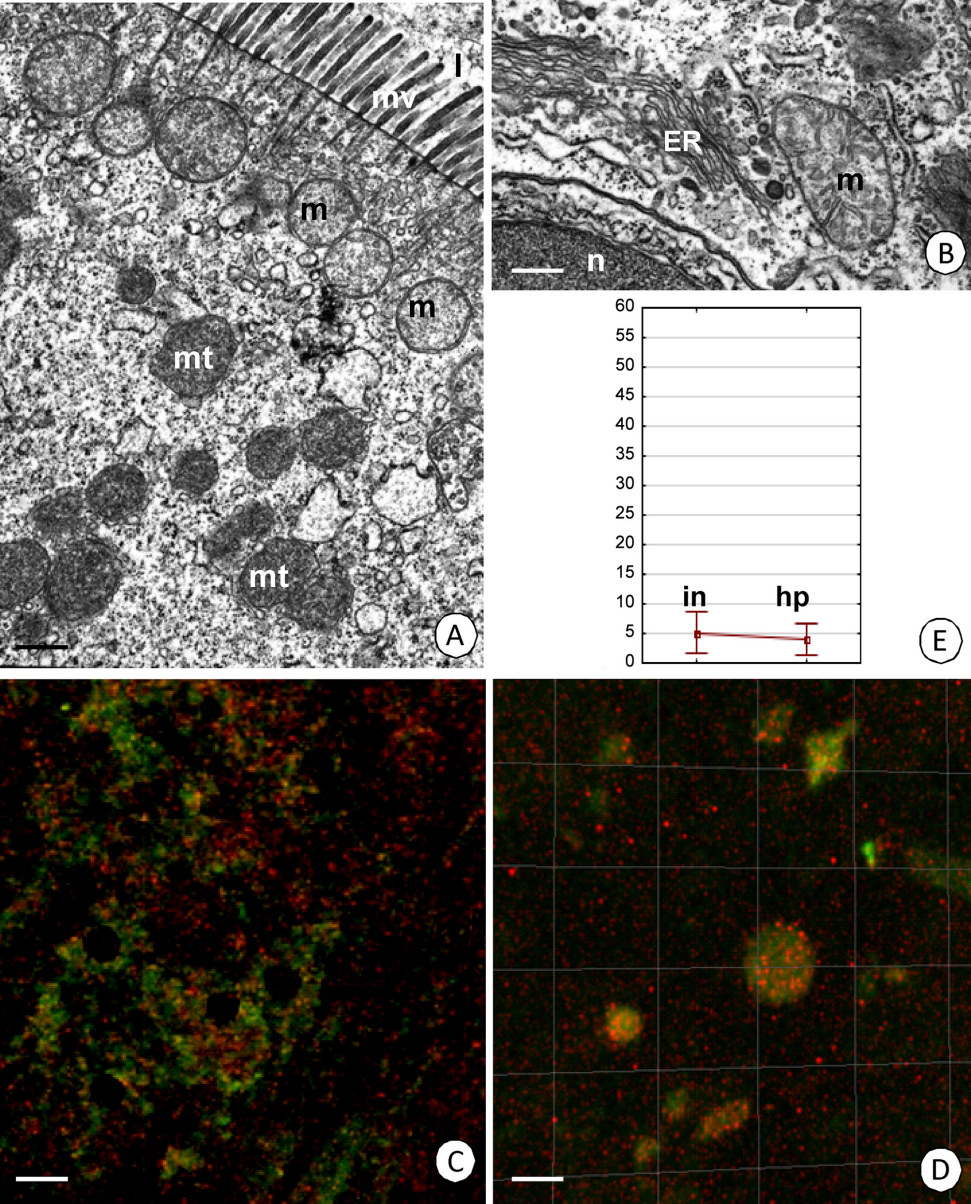
Mitochondrial potential in the *N*. *heteropoda* midgut epithelium. **(A)** Transformed mitochondria (mt) that are devoid of cristae and normal mitochondria (m) in the apical cytoplasm of the digestive cells of intestine. Microvilli (mv), midgut lumen (l). TEM. Bar = 0.6 μm. **(B)** Normal mitochondria (m) in the apical cytoplasm of the digestive cells of intestine. Cisterns of ER (ER), fragment of the nucleus (n). TEM. Bar = 0.4 μm. **(C-D)** Active mitochondria with a high membrane potential (red signals), inactive mitochondria with a low membrane potential (green signals). JC-1 cationic dye. Confocal microscope. **(C)** Bar = 10 μm. **(D)** Bar = 9 μm. **(E)** A diagrammatic representation of the average percentage of cells with depolarized mitochondria in the intestine (in) (5.1%) and hepatopancreas (hp) (3.9%).

In both of the organs that were examined, transformed mitochondria with electron-dense matrix were observed together with unchanged mitochondria (Fig 7A and 7B). They were distinguished in the cytoplasm of the D-cells in the entire intestine (Fig 7A) and the F- and B-cells of hepatopancreas. Application of the membrane-permeant JC-1 cationic dye indicated that active mitochondria that had a high membrane potential showed a red fluorescence, while green fluorescence of the monomeric form of the dye indicated the localization of inactive mitochondria that had a low membrane potential (Fig 7C and 7D, S5 Video 5, S6 Video). The average percentage of cells with depolarized mitochondria was low in both the intestine (5.1%) and the hepatopancreas (3.9%). There were no statistically significant differences in the level of this parameter between the organs that were analyzed (Table 5, Fig 7E).

**Table 5:** Percentage [%] of intestinal and hepatopancreatic epithelial cells with depolarized mitochondria in *N*. *heteropoda*.

	percentage [%]
	of cells with depolarized mitochondria
**intestine 1**	5.0
**intestine 2**	4.3
**intestine 3**	3.3
**intestine 4**	2.2
**intestine 5**	4.5
**intestine 6**	12.8
**intestine 7**	3.8
**hepatopancreas 1**	6.8
**hepatopancreas 2**	8.4
**hepatopancreas 3**	1.2
**hepatopancreas 4**	2.9
**hepatopancreas 5**	1.8
**hepatopancreas 6**	2.6
**hepatopancreas 7**	3.8

## Discussion

Cell death in multicellular organisms may occur as a natural process in which old cells die and are replaced by newly formed ones. It can also be activated by external factors that can affect cellular stress or even inflammation (e.g., heat, reactive oxygen species, toxic metals, xenobiotics, pathogens, starvation, lack of water, etc.) [3,4,14–16]. The best-known types of cell death that have been clearly described in the digestive system of arthropods are apoptosis, necrosis and autophagy [17–24]. These were classified as different because of their various morphological appearances [25].

In the species that was examined in this study, all of the described types of cell death, apoptosis, necrosis and autophagy, only concerned the D-cells in the intestine and B- and F-cells in hepatopancreas (Table 6). The process of cell death has also been detected in the F-, B- and R-cells of the digestive epithelia in some other crustaceans [26–29]. Regenerative cells (E-cells) in *N*. *heteropoda* were not affected by cell death. This is connected with the fact that D-, F and B-cells are fully developed cells of the midgut epithelium in the species that was examined here and they are responsible for the synthesis and secretion of digestive enzymes (D-cells, F-cells), or for the storage of the reserve material and secretion (B-cells) [13,30–33]. E-cells, which are the stem cells of the midgut, are responsible for the regeneration of the midgut [34,35]. In the intestine of *N*. *heteropoda*, they accumulate only in the anterior region where they are located individually between the basal regions of D-cells and have no contact with the intestine lumen. In the case of the hepatopancreas, E-cells are located in the distal zone of the hepatopancreatic tubules and form a blind-ended closure [13]. Therefore, only cells that take part in the digestion of nourishment gradually die.

**Table 6:** The localization of autophagy, apoptosis and necrosis in different regions of the midgut in *N*. *heteropoda*.

	Type of cells	Autophagy	Apoptosis	Necrosis
**Intestine anterior region**	*D-cells*	+	+	+
**Intestine anterior region**	*R-cells*	-	-	-
**Intestine posterior region**	*D-cells*	+	-	+
**Hepatopancreas distal zone**	*R-cells*	-	-	-
**Hepatopancreas medial zone**	*differentiating R-cells*	-	-	-
**Hepatopancreas proximal zone**	*F-cells*	+	+	+
**Hepatopancreas proximal zone**	*B-cells*	+	+	+

Up to now, the process of the cell death in the digestive epithelia of Crustacea has been called degenerative desquamation [28,36,37]. This process is revealed by cell lysis, nuclear degradation, pyknosis and cytoplasmic vacuolization. The neighboring cells gradually push the degenerating and damaged cells into the hepatopancreatic lumen. In some species, the degenerative desquamation appears during the molting cycle, so it occurs in a cyclic manner [33,38,39] as has been described for many insect species [40,41]. It can also be caused by many external factors, such as pathogens [28, 33, 36, 37, 42]. However, it is difficult to state whether the process of apoptosis or necrosis was observed during above-mentioned studies. We can only find sporadic data that the degenerative desquamation has been called a typical necrosis in Crustacea [29,33]. Additionally, the process of autophagy is poorly understood in this group of arthropods [20]. Therefore, our studies present the first precise description of apoptosis, autophagy and necrosis in this group of arthropods.

In animals, the cell shrinks and is gradually separated from adjacent cells during apoptosis. Simultaneously, the nucleus develops a lobular shape and prior to fragmentation has dense patches of chromatin. Eventually, the fragmented apoptotic cell is phagocyted by macrophags, neighboring cells or is degraded in the extracellular space when cells that are capable of phagocytosis are absent [21, 43–45]. The cytoplasmatic swelling, karyolysis, vacuolization and finally the lysis of all organelles and the entire cytoplasm is called necrosis (oncosis) [15,46]. However, autophagy is connected with the formation of structures that are classified as the phagophore, autophagosome, autolysosome and eventually, the residual body [47,48]. The ultrastructural alterations that appear in the epithelial cells in intestine and hepatopancreas during apoptosis, necrosis and autophagy in *N*. *heteropoda* are typical to those described above and in the midgut epithelia of other arthropods [4, 19, 20, 21, 22, 23, 24, 33, 40, 49].

We observed that while autophagy and necrosis were detected in the intestinal D-cells along the entire length of the intestine, apoptosis was only observed in the anterior region of the intestine where E-cells were detected (about 23% of the cells in this region showed signs of apoptosis). The correlation between the accumulation of E-cells and the activation of apoptosis in the anterior region of the intestine seems to be interesting. The relationship between cell proliferation and cell death (mainly apoptosis) plays an important role in tissue-size homeostasis maintenance. Their cooperation and the balance between them enable the number of cells to be controlled [2,50]. The results presented here confirm such a statement in the case of intestine. However, apoptosis is involved in the cell death of about 18% of cells (F- and B-cell together) in the hepatopancreatic tubules. As we described in our previous studies [13], the hepatopancreatic tubules show a distinct regionalization along their length–the distal zone with E-cells, the medial zone with differentiating cells and the proximal zone with fully developed F- and B-cells. In the latter, we observed that when the distance of F- and B-cells from the medial zone of the tubule was longer, their ultrastructure changed. Therefore, this region must have cells, which as a result of the functions they perform, must eventually die. In the digestive epithelia of many invertebrates the process of apoptosis is a natural process that is responsible for the neutralization of epithelial cells, when the cells are not able to fulfill their functions [4, 21, 43, 44, 45].

Necrosis is a form of cell death that is connected with cell injury and can be triggered by external factors or mechanical damage [51, 52, 53, 54]. To date, necrosis (called desquamation) has only been described in the hepatopancreas of decapods [29,33]. In *N*. *heteropoda* that was analyzed, necrosis was a sporadic process (about 4.8% of the D-cells in the intestine and 1.9% in the hepatopancreas) and it occurs along the entire length of the intestine and in the entire proximal zone of the hepatopancreatic tubules. Therefore, we can conclude that this process is accidental and is caused by the food masses that enter the intestine lumen as has been suggested for other crustacean species, *Eubranchipus grubii* [20].

The dominant type of the cell death that appears in the midgut epithelium of shrimp that were analyzed is autophagy (about 40% of the D-cells showed signs of autophagy), which is known to be a kind of cell death or pro-survival process. Autophagy can be activated by starvation or any external stressors [22–24,47,48,55]. It plays an important role in regulating the turnover and removal of organelles and proteins and protects the cell against any extracellular factors [14,20,21,48,56], or even the entire tissue/organ against degeneration in the case of a lack of regenerative cells [20]. Autophagy has been described as one of the mechanisms that prevent the midgut epithelial cells from degeneration [20, 24, 55]. In the case of species that do not have regenerative cells (E-cells) in their midgut epithelium, which are responsible for the self-renewal of cells, the midgut must survive as long as possible. Therefore, the process of autophagy is intensive [20]. Because of the fact that autophagy was detected along the entire length of the intestine and in the proximal zone of hepatopancreatic tubules, we can conclude that this process is probably responsible for separating degenerating organelles thus preventing the activation of the cell death similar to other crustaceans.

Mitochondria are multi-functional organelles that are not only connected with the production of ATP, the synthesis of steroids or reactive oxygen species (ROS), but also with programmed cell death [15,57]. They play an important role in the activation of cell death [10]. Measurements of the transmembrane mitochondrial potential (ΔΨm) are presumed to be a marker of any changes at the ultrastructural level that could lead to cell death before any visible changes at the higher levels of biological organization occur [10,12]. In freshwater shrimp *N*. *heteropoda* that were analyzed, cells with depolarized mitochondria were only detected in about 5% of D-cells of the intestine (along its entire length) and in about 3.9% of the epithelial cells in the hepatopancreatic tubules. Additionally, we observed mitochondria that were enclosed inside the autophagosomes. A selective degradation of organelles can occur during autophagy [47,48]. Hence, mitophagy is treated as a type of selective autophagy [58]. It promotes the degradation of mitochondria and eventually protects the entire cell against the cellular degeneration that is caused by mitochondrial dysfunction [59]. In the species that was analyzed here, degenerating mitochondria were neutralized in the autophagosomes, and therefore the number of cells with depolarized mitochondria was quite low and eventually, the process of apoptosis or necrosis could not be activated. However, the results that were obtained will be important during studies on the impact of stressors for the functioning and activation of cell death. The results on specimens that live without any stressors could be treated as the results of the control group during our further studies.

## Conclusions

The results of these studies showed that in freshwater shrimp *N*. *heteropoda* that were analyzed: (a) apoptosis, necrosis and autophagy were detected in the midgut epithelium; (b) they involve only the fully developed cells of the midgut epithelium that have contact with the midgut lumen; (c) ultrastructural alterations of midgut epithelial cells during apoptosis, necrosis and autophagy are typical as those described in the midgut epithelia of other arthropods; (d) there is a distinct correlation between the accumulation of E-cells and the activation of apoptosis in the anterior region of the intestine; (e) necrosis is an accidental process and (f) autophagy is probably responsible for the neutralization of degenerating organelles (e.g., mitochondria) thus preventing the activation of cell death; (g) the low number of cells with depolarized mitochondria was caused by intensive autophagy (mitophagy).

## Materials and Methods

### Materials

The studies were performed on adult males and females of the freshwater shrimp *Neocaridina heteropoda* (Crustacea, Malacostraca, Decapoda). The specimens were obtained from local shrimp breeders and kept in 30-liter aquaria in the laboratory (no specific permissions were required for breeding). The environmental conditions in the aquaria were strictly controlled, i.e. temperature of 24°C, pH of 7 and total water hardness of 15^0^d. The *N*. *heteropoda* specimens were fed with food that is produced for freshwater shrimps that consists mainly of vegetable by-products, algae and fish by-products. The principles of laboratory animal care were followed, as well as specific national laws where applicable.

### Methods

#### Light and transmission electron microscopy

Adult specimens of *N*. *heteropoda* were decapitated (15 females and 15 males) and fixed with 2.5% glutaraldehyde in a 0.1 M sodium phosphate buffer (pH 7.4, 4°C, 2h), postfixed in 2% osmium tetroxide in a 0.1 M phosphate buffer (4°C, 1.5 h) and dehydrated in a graded series of concentrations of ethanol (50, 70, 90, 95 and 4x100% each for 15 min) and acetone (15 min). Eventually, the material was embedded in epoxy resin (Epoxy Embedding Medium Kit; Sigma). Semi- (0.8 μm thick) and ultra-thin (70 nm) sections were cut on a Leica Ultracut UCT25 ultramicrotome. Semi-thin sections were stained with 1% methylene blue in 0.5% borax and observed using an Olympus BX60 light microscope. After staining with uranyl acetate and lead citrate, ultra-thin sections were examined using a Hitachi H500 transmission electron microscope. Ultrathin sections were used in order to count the number of cells that had signs of necrosis or autophagy in relation to the total number of midgut epithelial cells. The percentage of necrotic or autophagic cells was determined by randomly counting cells in the intestine and hepatopancreas of 3 adult specimens. Significance of differences in the levels of percentage of necrotic cells and autophagic cells between intestine and hepatopancreas was assessed with the Student’s t-test, p<0.05.

#### Acid phosphatase staining–detection of lysosomes and autolysosomes (TEM)

The midguts (intestine and hepatopancreas) from two specimens were fixed in Karnovsky’s fixative (2h, 4°C), washed in a cacodylate buffer 0.1M (15 min, RT), 0.2M Tris-Maleate (10 min, RT) and incubated in the incubation medium– 0.2M Tris-Maleate buffer, 0.1M sodium β-glycerophosphate and 0.2M lead citrate (2h, 37°C). After washing the material with Tris-Maleate (2 x 15 min, RT), it was postfixed in 1% osmium tetroxide and prepared according to the standard method for TEM. The material was then analyzed using a Hitachi H500 transmission electron microscope.

#### Cryosections

Adult specimens of *N*. *heteropoda* (10 males and 10 females) without fixation were embedded in a tissue-freezing medium (Jung). Cryostat sections were cut (5 μm thick) and mounted on slides.

#### Acid phosphatase staining–detection of lysosomes and autolysosomes (light microscope)

After washing in Tris-buffered saline (TBS) (5 min, RT) and a 0.1N sodium acetate-acetic acid buffer (pH 5.0–5.2, RT), cryosections were incubated in a 0.1N sodium acetate-acetic acid buffer (pH 5.0–5.2, 1,5h, 37°C) containing 0.01% naphtol phosphate AS-BI, 2% *N-N*-dimethylformamide, 0.06% Fast Red Violet LB, 0.5mM MnCl_2._ Negative controls were performed by omitting the specific naphtol phosphate AS-BI substrate. The material was analyzed using an Olympus BX60 light microscope.

#### LysoTracker Red (LTR) staining–autophagosomes and autolysosomes labelling

This red-fluorescent dye selectively accumulates in acidic organelles and can be used to investigate lysosomes and autolysosomes. The intestine and hepatopancreas isolated from animals body without fixation were incubated in the dark for 15 min in 2.5 mM LysoTracker Red DND-99 (Molecular Probes, L 7528) diluted in 500 ml of PBS (Phosphate-buffered saline) at RT. Next, the material was washed several times with PBS. The nuclei were stained for 10 min in 1 mg/ml Hoechst 33342 diluted in PBS and washed several times in PBS. The slides were analyzed using an Olympus FluoView FV1000 confocal microscope. Excitation at 543 nm was provided by an argon/krypton laser.

#### TUNEL assay–detection of DNA fragmentation during apoptosis

Terminal deoxynucleotidyl transferase dUTP nick end labeling (TUNEL) is the method that is used to investigate the DNA fragmentation that occurs during apoptosis. The intestine and hepatopancreas isolated from animals body without fixation were washed in TBS (3 x 5 min) and stained with a TUNEL reaction mixture (*In Situ* Cell Death Detection Kit, TMR red, Roche) (60 min at 37°C in the dark). After washing in TBS, the material was analyzed using an Olympus FluoView FV1000 confocal microscope with 60×/NA 1.35 objectives. Z-stack images were generated using a 405nm laser for Hoechst 33342 dyes and 568nm for TMR red dye (TUNEL reaction mixture). 3D data sets were analyzed as volume-rendered data sets using Imaris (custom software developed by Bitplane Scientific Software, Zurich, Switzerland). For statistical analysis all cells were segmented using the surface modeling that is available in Imaris for Hoechst 33342 dye. The mean fluorescence value for TMR red dye was calculated for each segmented cell. The arbitrary mean intensity threshold for the TMR red dye was used to determine the number of stained cells. As a result, we calculated the mean percentage of the stained cell population. Negative controls without terminal deoxynucleotidyl transferase (TdT) were prepared according to the labeling protocol (*In Situ* Cell Death Detection Kit, TMR red, Roche). Statistical analyses were performed using the R (ver. 3.0.2) statistical environment. Normality was checked by the Shapiro-Wilk test. Significance of differences in the levels of percentage of apoptotic cells between intestine and hepatopancreas in TUNEL assay was assessed with the Student’s t-test, p<0.05.

#### Preparation of cell suspension

Dissected organs (hepatopancreas and intestine that were isolated from five specimens) were crushed mechanically and suspended in 100 μL PBS (pH 7.4). Then, using 0.05% trypsin in EDTA solution with 0.01% collagenase II, enzymatic isolation was carried out for 10 min at 37°C. The cells were suspended in DMEM low-glucose medium (1 g/L) and incubated at 37°C. The cell suspension was washed using centrifugation at 1500 rpm for five minutes and the precipitate was suspended in 100 μL of PBS buffer.

#### Quantitative assessment of cells with depolarized mitochondria

JC-1 (5,5ʹ,6,6ʹ-tetrachloro-1,1ʹ,3,3ʹ-tetraethyl-benzimidazolyl-carbocyanine iodide) is a membrane-permeant cationic dye that is widely used in order to monitor the mitochondria in cell death studies. Changes in mitochondrial transmembrane potential (ΔΨm) were monitored using JC-1 cationic dye whose accumulation in mitochondria is dependent on the magnitude of mitochondrial potential. JC-1 differentiates cells with a high mitochondrial potential (orange fluorescence; polarized mitochondria) and a low mitochondrial potential (green fluorescence; depolarized mitochondria) (Salvioli et al., 1997). The intestine and hepatopancreas were isolated from 7 animals body. The cell suspension obtained from these organs without fixation was incubated in the dark with 5 μL of 1.5 mM JC-1 solution in DMSO (99.97%, H_2_O < 0.1%) for 15 minutes at room temperature. The cells were analyzed using flow cytometry (Coulter Instrument EPICS XL MLC) with a 488 nm argon laser, using the MXP software Beckman Coulter program and the results were presented as the percentage of cells with depolarized mitochondria. Statistical analyses were performed using the STATISTICA 10.0 software package (StatSoft, Inc. (2010) version 10.0. http://www.statsoft.com). Normality was checked by the Kolmogorov-Smirnov test. The data were tested for homogeneity of variance using Levene’s test of equality of error variances. Significance of differences in the levels of percentage of cells with depolarized mitochondria between intestine and hepatopancreas was assessed with the Student’s t-test, *p<0*.*05*.

#### JC1 staining–mitochondrial membrane potential for confocal microscopy

The prepared intestine and hepatopancreas isolated from animals body were incubated in the dark with 5 μL of 1.5 mM JC-1 solution in DMSO (99.97%, H_2_O < 0.1%) for 15 minutes at room temperature without fixation. The material was visualized using an Olympus FluoView FV1000 confocal microscope.

## Supporting Information

S1 Abstract Congress of Molecular Biology, Poland (in Polish). (TIF)

S1 Author Summary (DOC)

S1 Video 3D representation of the localization of lysosomes in the intestine of *N*. *heteropoda*. Autolysosomes (red), nuclei (blue). LysoTracker Red and Hoechst 33342 staining. Confocal microscope.
(MPEG)

S2 Video 3D representation of the localization of lysosomes in the hepatopancreas of *N*. *heteropoda*. Autolysosomes (red), nuclei (blue). LysoTracker Red and Hoechst 33342 staining. Confocal microscope.
(MPEG)

S3 Video 3D representation of apoptotic cells in the intestine of *N*. *heteropoda*. Nuclei of apoptotic cells (red), nuclei (blue). TUNEL assay and Hoechst 33342 staining. Confocal microscope.
(MPEG)

S4 Video 3D representation of apoptotic cells in the hepatopancreas of *N*. *heteropoda*. Nuclei of apoptotic cells (red), nuclei (blue). TUNEL assay and Hoechst 33342 staining. Confocal microscope.
(MPEG)

S5 Video 3D representation of the mitochondrial transmembrane potential in the intestine of *N*. *heteropoda*. Localization of mitochondria with a low potential (green), mitochondria with a high potential (orange). JC-1 staining. Confocal microscope.
(MPEG)

S6 Video 3D representation of the mitochondrial transmembrane potential in the hepatopancreas of *N*. *heteropoda*. Localization of mitochondria with a low potential (green) and mitochondria with a high potential (orange). JC-1 staining. Confocal microscope.
(MPG)
