# Supplementary figures and images for: Cell Death in the Epithelia of the Intestine and Hepatopancreas in Neocaridina heteropoda (Crustacea, Malacostraca)
Source: PLoS One. 2016 Feb 4;11(2):e0147582. doi: 10.1371/journal.pone.0147582 (PMC4741826; doi:10.1371/journal.pone.0147582)

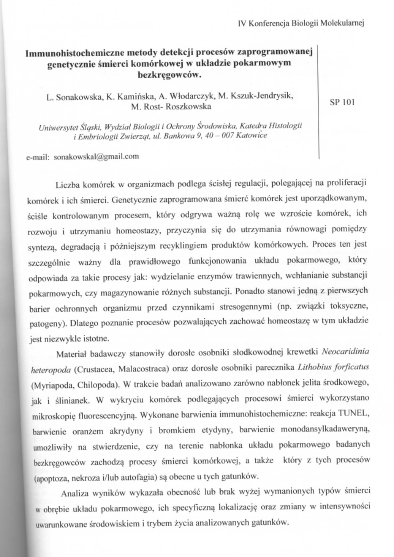

Supplement: S1 Abstract — (TIF) [file pone.0147582.s001.tif]
