# Supplementary material for: Cell Death in the Epithelia of the Intestine and Hepatopancreas in Neocaridina heteropoda (Crustacea, Malacostraca)
Source: PLoS One. 2016 Feb 4;11(2):e0147582. doi: 10.1371/journal.pone.0147582 (PMC4741826; doi:10.1371/journal.pone.0147582)
Supplement: S1 Author Summary — (DOC) [file pone.0147582.s002.doc]

**Author Summary**

The programmed cell death (PCD) plays an important role in maintaining the homeostasis of tissues, organs and finally, the entire organism. Among all described types of the programmed cell death, apoptosis, necrosis and autophagy have been detected in invertebrates digestive system. The endodermal region of the digestive system (called as the midgut) in some of Crustacea is composed of an intestine and a hepatopancreas. As the material for our study we chose freshwater shrimp which is widely bred all over the world, and we presented the precise structure and ultrastructure of its midgut during our previous work. In this work we used transmission electron microscopy to present the course of apoptosis, necrosis and autophagy in the intestine and hepatopancreas of the species examined. Light and confocal microscopes let us to reveal the localization and the number of apoptotic, necrotic and autophagic cells in both regions of the midgut. Additionally, we presented the results of quantitative assessment of cells with depolarized mitochondria. The cell death have been described only in D-cells in intestine and F- and B-cells in hepatopancreatic tubules, while E-cells did not die. Here we also present the first description of the precise course of apoptosis, necrosis and autophagy at the ultrastructural level in Crustacea.

**Blurb**

1. The cell death appears in the intestine and proximal zone of hepatopancreatic tubules of the midgut in *Neocaridina heteropoda.*
2. The localization of apoptotic, necrotic and autophagic cells in epithelial cells in intestine and hepatopancreas with the emphasis on their functions has been shown.
